# ﻿Diversity, abundance and distribution of caddisfly (Insecta, Trichoptera) families in relation to environmental parameters across freshwater streams in Singapore

**DOI:** 10.3897/zookeys.1263.147968

**Published:** 2025-12-10

**Authors:** Yee Qi Chan, Bryna Jia Ying Liang, Yuchen Ang, John C. Morse, Yixiong Cai, Darren C. J. Yeo

**Affiliations:** 1 Department of Biological Sciences, National University of Singapore, 16 Science Drive 4, Singapore 117558, Singapore; 2 Lee Kong Chian Natural History Museum, National University of Singapore, 2 Conservatory Drive, 117377, Singapore; 3 Department of Plant & Environmental Sciences, Clemson University, Clemson, South Carolina 29634-0310, USA; 4 National Parks Board, Singapore, 1 Cluny Road, Singapore 259569, Singapore

**Keywords:** Bioindicator, biomonitoring, caddisflies, ecology, ecosystem services, insects, macroinvertebrate, urbanisation

## Abstract

In land-scarce Singapore, where fresh water is a critical resource, Trichoptera communities can serve as a biomonitoring tool, yet are poorly known. This study seeks to address this gap by establishing a preliminary understanding of trichopteran diversity and distribution in Singapore’s freshwater streams. From October 2023–January 2024, 11 stream sites across forested nature reserves (*N* = 5), buffer parks (*N* = 4), and urban areas (*N* = 2) were surveyed for trichopteran larvae. Only eight sites (four in forest streams, four in buffer streams) yielded Trichoptera, totalling 107 larval specimens comprising six families (Calamoceratidae, Ecnomidae, Hydropsychidae, Leptoceridae, Odontoceridae, and Polycentropodidae). Leptoceridae were most abundant, Ecnomidae were the rarest, and Hydropsychidae were the most widely distributed. In this study, Trichoptera were absent from the urban streams, which had greater depth and total dissolved solids, but similar Trichoptera assemblages and environmental parameters were recorded in both forest and buffer streams. Although differences between the latter two stream types were not statistically significant, buffer streams had the highest abundance and taxonomic richness. Comparison with past literature also reveals differences in recorded Trichoptera diversity, thus, this study presents an updated record for Trichoptera in Singapore’s streams. These findings further add to a baseline for future biomonitoring, research, and informing long-term freshwater conservation efforts in Singapore.

## ﻿Introduction

Trichoptera, also known as caddisflies, comprise one of the world’s most diverse orders of insects with aquatic larvae, with over 17,000 species currently described globally from all biogeographical regions save Antarctica (Morse et al. 2019). Trichoptera are amphibiotic, with immature stages inhabiting aquatic environments and adult stages occupying terrestrial habitats near water bodies, linking ecosystems and enhancing functional diversity (Morse et al. 2019). Trichoptera also display great functional diversity in their larval stages, having a wide variety of feeding modes and retreat forms in freshwater systems (Holzenthal et al. 2015; Morse et al. 2019). Similar to other macroinvertebrates, Trichoptera functional diversity is also greatly shaped by environmental factors including water temperature (as affected by altitude, latitude, riparian shade, etc.), water flow rate, dissolved substances, and substrate type (Hynes 1970; Huryn and Wallace 1988; Lamouroux et al. 2004; Hughes 2006). As a result, trichopteran assemblages fulfil diverse niches within freshwater ecosystems, generating many essential ecosystem services (Morse et al. 2019). Consequently, Trichoptera are important bioindicators of water quality and environmental stress, especially given their wide range of pollution tolerances (Blakely et al. 2014; Kalaninová et al. 2014; Morse et al. 2019). Freshwater life stages of many caddisfly species are highly susceptible to physico-chemical changes in streams, which are increasingly attributed to urbanisation and anthropogenic impacts (Pickett et al. 2011). Therefore, the long-term monitoring of Trichoptera populations is crucial in the management and conservation of freshwater habitats and for the evaluation of urbanisation impacts (Wiederkehr et al. 2020).

While male adults are usually required for identification to species level, larval characteristics are sufficient for identification of most of the 30 Southeast Asian trichopteran families (Morse 2025). The Oriental Region, where Singapore is situated, has the highest known diversity of Trichoptera among all biogeographical regions, with 5854 species recorded as of 2019 (Morse et al. 2019). Yet despite the increasing focus on the diversity and importance of Trichoptera within the academic and biomonitoring communities, no published systematic work on Singapore species exists to date. Little is also known about trichopteran distribution in Singapore, apart from families and genera discovered in a dedicated survey of macroinvertebrates in Singapore’s last remnant freshwater swamp forest (Ho et al. 2018) and a study of macroinvertebrate assemblages across forest, buffer, and urban streams (Wiederkehr et al. 2020).

To deepen current understanding of Trichoptera in Singapore, this study aimed to discover and describe Singapore’s Trichoptera diversity and distribution in freshwater streams along an urbanisation gradient. Specifically, this study determined and compared the taxonomic diversity of trichopteran larvae (1) across forest, buffer, and urban streams, and (2) in relation to differences in environmental conditions along the continuum of stream types. Study findings contribute to global records of Trichoptera, furthering the understanding and identification of this diverse taxon while also informing local conservation and management of Singapore’s freshwater streams and Trichoptera assemblages.

## ﻿Material and methods

### ﻿Sampling sites

**Figure 1.**
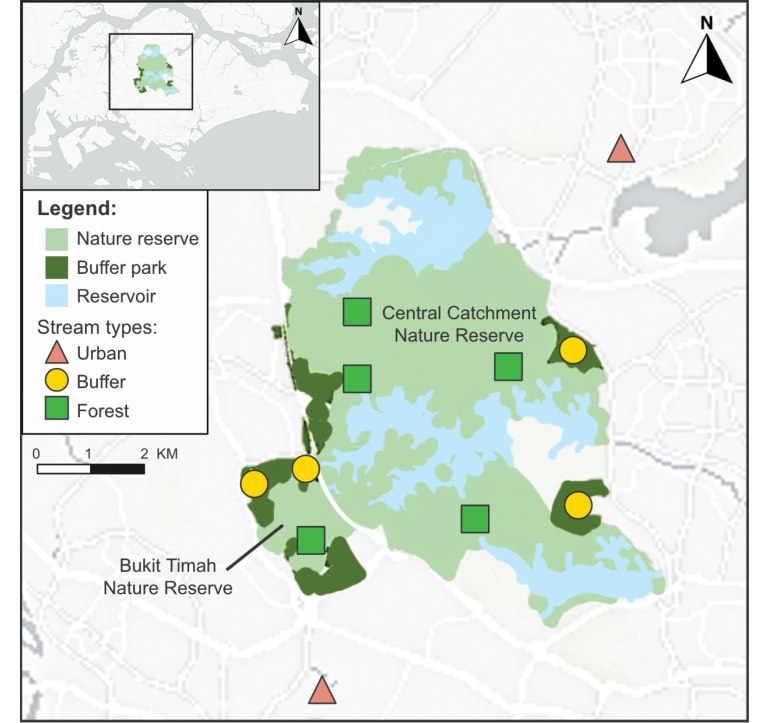
Map showing 11 sampling sites included in the present study. These include sites on five forest streams (green square), four buffer streams (yellow circle), and two urban streams (red triangle). Sites are shown in relation to the Central Catchment Nature Reserve and the Bukit Timah Nature Reserve, which are located in the central part of Singapore (inset).

Eleven sites (Suppl. material 1: table S1) across freshwater streams in Singapore were each sampled once for Trichoptera larvae during the Northeast Monsoon months of October 2023–January 2024 in the mornings (0930h–1330h). These sites were categorised into three stream types (forest, buffer, and urban) depending on where they were located, following the geographical boundaries of nature reserves and buffer parks determined by the Singapore National Parks Board (NParks). Given that nature reserves contain Singapore’s remaining primary forest patches as well as native flora and fauna, they are of highest conservation priority and most protected from urbanisation impacts (NParks 2025). Buffer parks border the nature reserves, thus buffering the latter from urbanisation impacts while providing spaces for recreation (NParks 2025). Urban or open-country areas are not managed for the purpose of biodiversity conservation, hence these three stream types generally reflect an urbanisation gradient. Accordingly, forest streams are situated in nature reserves, buffer streams in buffer parks and urban streams in urban or open-country areas not immediately adjacent to the nature reserves. This study sampled five forest streams in Central Catchment Nature Reserve (excluding Nee Soon Swamp Forest; NSSF) and Bukit Timah Nature Reserve, four buffer streams in buffer parks like Thomson Nature Park and Windsor Nature Park, and two urban streams in the residential towns of Yishun and Clementi (Fig. 1).

### ﻿Collection of specimens and environmental parameters

At each stream site, a 10-m longitudinal transect was measured and environmental parameters were collected at 0 m, 5 m, and 10 m. These included temperature (°C), dissolved oxygen (DO; mg/L), conductivity (μS/cm), total dissolved solids (TDS; mg/L), salinity (ppt), and pH, all of which were measured using a multi-meter probe (YSI® Professional Plus; Xylem Analytics). Wetted width (cm) and water depth (cm) were measured using a 30-cm wooden ruler, with three readings taken for water depth across the width of the stream – one near each stream edge and one in the centre of the stream. The average of the three readings taken in each transect at 0 m, 5 m, and 10 m was then obtained. General hardness of the water was determined using a hardness test (Salifert®) and surface flow rate (km/h) was determined either using a flow meter (Flowatch®; JDC Electronics SA) or a stopwatch (timings were recorded for a floating object to travel 1 m). Three readings for surface flow were taken in each transect at 0 m, 5 m, and 10 m, and the average of the three readings was obtained. Finally, canopy cover (%) was recorded using a spherical densiometer (Spherical Crown Densiometer® Concave Model C; Forestry Suppliers).

The kick and tray-netting method (Ho et al. 2018) was employed in each 10-m transect using kick nets (36 × 30 cm, 500 μm mesh size) for sampling stream macroinvertebrates and was standardised across sites. Substrate at both the sides and centre of the stream were disturbed vigorously by two field surveyors concurrently for five minutes, sampling the entire length of the 10-m transect from downstream to upstream. Dislodged organisms and material were collected by placing the kick net downstream of where field surveyors had disturbed the water and sediment. After removal of large debris, the remaining collected material from the kick nets was passed through two sieves, first through a mesh size of 2.0 mm and then 250 μm. Any vertebrates or decapods collected were returned to the stream, and any visible Trichoptera larvae and other macroinvertebrates were stored separately in labelled tubes containing 75% ethanol. Remaining material in the 250 μm sieve was then bagged, labelled, and kept separately with ice for transportation and storage.

### ﻿Sample processing and Trichoptera larvae identification

Upon returning to the laboratory, the bagged material was washed and stored in 96% ethanol (Suppl. material 1: fig. S1). This material was subsequently sorted, without magnification, for Trichoptera larvae and other macroinvertebrates (Suppl. material 1: fig. S2), which were verified under a stereo microscope (Leica EZ4, 35× magnification) and then stored with those collected at the same site in 75% ethanol. For sites where no Trichoptera were detected during sampling, a subsample comprising 30% of collected material was sorted without magnification to confirm the absence of Trichoptera and the remaining material was not sorted. Finally, all collected Trichoptera larvae were removed from cases, if present, (but retained with the specimen, Suppl. material 1: fig. S3) and thereafter identified to family level (Suppl. material 1: fig. S4) under a stereo microscope (Leica M205 C, 160× magnification) using a Trichoptera identification key developed for the Malaysian region (Morse 2004).

### ﻿Data analysis

All statistical analyses were performed using R version 4.2.2 (R Core Team, 2022) at 5% significance level.

### ﻿Diversity, richness and abundance of Trichoptera assemblages

Family abundance and richness of the Trichoptera assemblage at each site were first determined and the relative abundance (*p_i_*) of each family (*i*) was calculated. The Shannon Index (*H*’) was then computed as a measure for diversity using the following equation:

Shannon Index (*H*’) = - ∑[(*p_i_*) × ln(*p_i_*)]

Comparison of abundance, richness, and Shannon Index was made across stream types. Differences were tested for statistical significance using Mann-Whitney U-test given the small sample sizes of buffer (*N* = 4) and forest streams (*N* = 5).

### ﻿Comparison of Trichoptera assemblages and environmental parameters across stream types

Trichoptera assemblages were compared across stream types by conducting non-metric dimensional scaling (NMDS) using the Bray-Curtis distance measure to identify any clustering of families or sites. Since Trichoptera specimens were only found in buffer streams and all forest streams except one, any variation between buffer and forest streams was then tested for statistical significance via permutational multivariate analysis of variance (PERMANOVA) using the *adonis2* function with the Bray-Curtis distance measure. Furthermore, since the Bray-Curtis distance measure cannot compute sites with no specimens, ‘0’ was replaced with a very small value. Environmental parameters were compared across stream types via a Principal Component Analysis (PCA), in which the environmental data used were site averages of three readings per transect. Differences in environmental parameters were then tested for statistical significance using the Mann-Whitney U-test, given the small sample sizes of buffer (*N* = 4) and forest streams (*N* = 5).

### ﻿Relating Trichoptera distribution to environmental parameters across sites

To relate Trichoptera distribution with environmental parameters across sites, a Constrained Analysis on Principal Coordinates (CAP) was done using the *vegan* R package with Bray-Curtis distance (Dixon, 2003) to identify any environmental correlates determining Trichoptera family distribution. Overlapping environmental vectors revealed through a preliminary CAP biplot were removed to obtain better separation of vectors in the final biplot. PERMANOVA was then conducted using the *adonis2* function with the Bray-Curtis distance measure to determine the statistical significance of the associations between environmental parameters and family distribution across sites.

## ﻿Results

### ﻿Trichoptera assemblages across sites and stream types

The six families of Trichoptera collected comprise Leptoceridae, Hydropsychidae, Odontoceridae, Calamoceratidae, Polycentropodidae, and Ecnomidae. None were found in urban streams, five families were found in forest streams, and Calamoceratidae occurred only in buffer streams along with the five families also found in forest streams (Table 1). Across sites, Leptoceridae were the most abundant with 54 individuals, with the highest relative abundance in both forest and buffer streams at 58.3% and 46.5%, respectively. In contrast, Ecnomidae were the least abundant, with two individuals across all sites, contributing 1.9% of relative abundance in total.

**Table 1.** Checklist of Trichoptera families with absolute and relative abundances across sampled buffer and forest streams.

Family	Buffer		Forest		Total abundance	Total relative abundance (%)
	Abundance	Relative Abundance (%)	Abundance	Relative abundance (%)		
Leptoceridae	33	46.5	21	58.3	54	50.5
Hydropsychidae	20	28.2	9	25	29	27.1
Calamoceratidae	8	11.3	0	0	8	7.5
Odontoceridae	7	9.9	4	11.1	11	10.3
Polycentropodidae	2	2.8	1	2.8	3	2.8
Ecnomidae	1	1.4	1	2.8	2	1.9
Total	71	100	36	100	107	100

**Figure 2.**
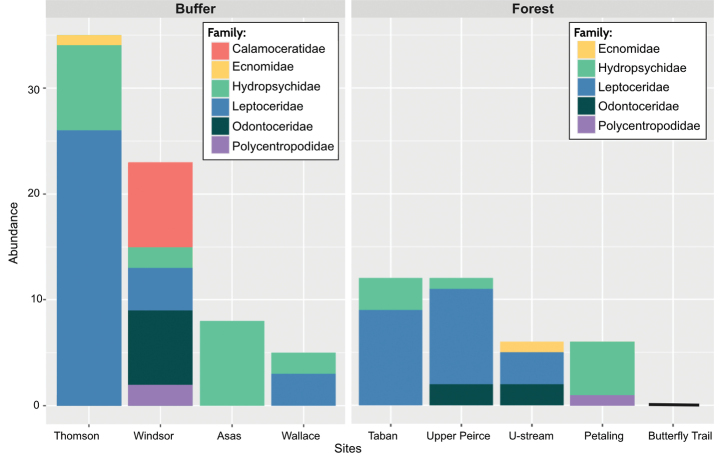
Stacked barplots of Trichoptera family composition, richness, and abundance at buffer and forest stream sites.

When comparing Trichoptera assemblages across individual forest and buffer stream sites (Fig. 2), all four buffer streams had Trichoptera, while only four out of five forest streams had Trichoptera. The highest Trichoptera abundance and richness were recorded in buffer streams, which also had the highest variability in abundance (5–35 individuals per site) and richness (1–5 families per site). In comparison, forest streams with Trichoptera had less variation in both abundance (6–12 individuals per site) and richness (2 or 3 families per site). Comparing all six Trichoptera families, Hydropsychidae were the most widespread, with specimens collected at seven sites, followed by Leptoceridae at six sites (Fig. 2). Calamoceratidae were the least widespread, occurring at only one buffer stream site (Fig. 2).

Using NMDS to compare Trichoptera assemblages across stream types (Suppl. material 1: fig. S5), the 95% confidence ellipses of buffer and forest streams overlap, and there is no statistically significant difference between Trichoptera assemblages across buffer and forest streams (*p*-value = 0.845). Further analysis revealed higher Trichoptera diversity, family richness, and abundance in buffer streams than in forest streams (Suppl. material 1: fig. S6), but again, the differences were not statistically significant.

### ﻿Environmental parameters across stream types

**Figure 3.**
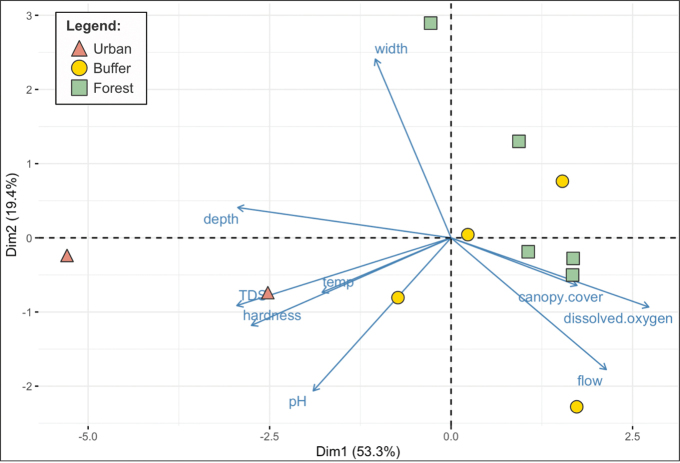
Principal component analysis (PCA) plot of environmental parameters. Vectors (blue) for urban (red), buffer (yellow), and forest (green) stream sites are shown, accounting for 72.7% of total variance. PCA axis 1 (Dim1) and axis 2 (Dim2) account for 53.3% and 19.4% total variance, respectively.

The PCA of environmental parameters collected across all three stream types reveals separation of urban sites from buffer and forest sites along PCA axis 1 (Dim1) and axis 2 (Dim2), which accounts for 72.7% of the total variation (Fig. 3). Dim1 appears to be associated with wetted depth, TDS, hardness, pH, dissolved oxygen, and flow, accounting for 53.3% of total variation. On the other hand, Dim2 is strongly associated with width and accounts for a further 19.4% of total variation.

The urban sites are situated on the left, driven mostly by greater depth, TDS, hardness, and pH. In comparison, the buffer and forest sites are situated more toward the right and this distinction from urban sites is driven by higher canopy cover, dissolved oxygen, and greater flow rate. Additionally, buffer and forest sites overlap, varying in terms of wetted width. Comparison of collected environmental parameters between buffer and forest streams reveals higher hardness, DO, conductivity, TDS, salinity, count, and population density in buffer streams than in forest streams, which are more acidic (Suppl. material 1: table S2). These differences, however, were not statistically significant (*p*-value > 0.05).

### ﻿Relating Trichoptera diversity to stream environmental parameters

**Figure 4.**
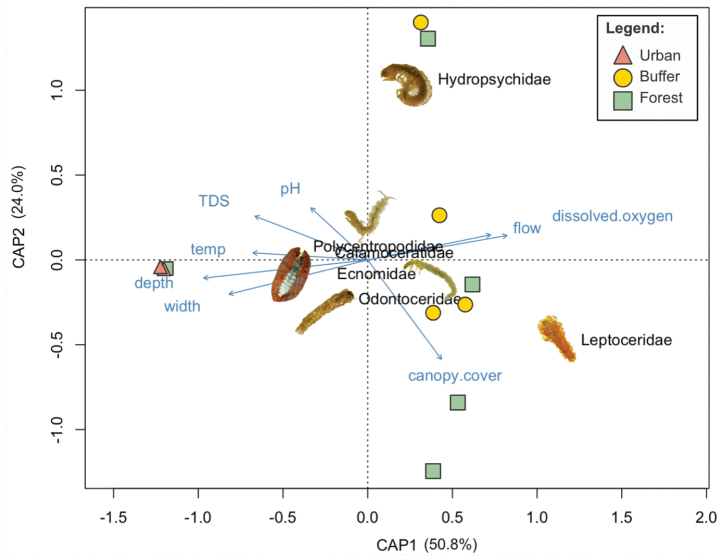
A Constrained Analysis on Principal Coordinates (CAP) biplot. Urban (red), buffer (yellow), and forest (green) stream sites are shown with environmental parameters represented by vectors (blue) and the six Trichoptera families collected. CAP1 and CAP2 account for 50.8% and 24.0% of variance, totalling 74.8% of accounted variance.

The CAP biplot (Fig. 4), accounting for 74.8% of total variance, shows a separation of sites without and with Trichoptera. Three sites without Trichoptera (two urban, one forest) are situated in the left half of the plot, while the four buffer and four forest streams with Trichoptera overlap in the right half of the plot, showing no distinct clustering (Fig. 4). CAP1 explains 50.8% of total accounted variance, and appears to be associated with flow rate, dissolved oxygen, temperature, depth, width, and TDS. Canopy cover and pH appear to be associated with CAP2, which explains 24% of total accounted variance.

Hydropsychidae appears to be associated with lower canopy cover, higher flow rate, dissolved oxygen, TDS, and pH; Leptoceridae with higher canopy cover, TDS and pH (Fig. 4). Polycentropodidae, Calamoceratidae, and Ecnomidae are clustered near the centre of the biplot and appear to be associated with greater depth and width, higher temperature, as well as lower flow and dissolved oxygen (Fig. 4). Among all eight environmental parameters included in the CAP, further statistical analysis confirmed that depth (*p*-value = 0.036) and flow (*p*-value = 0.029) were significant in explaining community differences.

## ﻿Discussion

### ﻿Urbanisation gradient

The taxonomic diversity of trichopteran larvae varied across forest, buffer, and urban streams. In relation to stream environmental conditions, buffer streams had the highest Trichoptera diversity among all sampled stream types in this study. Along this urbanisation gradient comprising a continuum of stream types, urban, buffer, and forest streams experience different types and extents of urbanisation impacts (Pickett et al. 2011), which often result in distinct physico-chemical properties, productivity, and macroinvertebrate community across stream types (Urban et al. 2006; Wiederkehr et al. 2020).

In the present study, stream types were determined based on the geographical designation of forested nature reserves and buffer parks (forest edges adjacent to nature reserves) by NParks (2024). Consequently, these streams receive different degrees of protection against disturbance and anthropogenic impacts, depending on how the surrounding area is managed (Wiederkehr et al. 2020). Urban streams in this study are in urban, open-country areas not immediately adjacent to the nature reserves that are not specifically managed or monitored, while forest streams likely receive the most protection due to strict regulations pertaining to access and the use of nature reserves for biodiversity conservation (NParks 2015). Buffer parks fall in the middle of this spectrum, being situated at forest edges, serving the fundamental purpose of buffering urbanisation and anthropogenic impacts on the core biodiversity areas, which are within the larger nature reserves (NParks 2015). Hence, streams within these buffer parks likely experience more disturbance than those in nature reserves, but less disturbance compared to urban streams, which often experience flash floods as well as high pollutant and allochthonous input from surface runoff (Delong and Brusven 1994).

**Figure 5.**
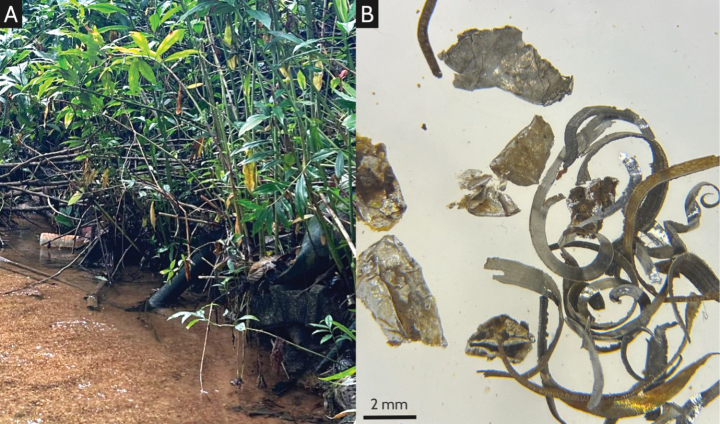
Trash observed in the present study. **A.** A bottle, a pipe, and a cement block in Asas stream; **B.** Metal pieces in the sediment collected from the stream in Thomson Nature Park.

The disturbance that buffer parks experience can be further discussed in both a historical and current context. Buffer parks are often situated near past settlements or in areas previously transformed by human activities (e.g., naturally occurring streams were redirected, straightened, canalised, etc.; Ong et al. 2023) and trash, such as broken pots and cement blocks, were found at some buffer stream sites along with finer metal pieces in the sediment (Fig. 5). As a result of this historical disturbance, active restoration efforts were undertaken prior to the opening of these buffer parks (Ong et al. 2023), with the first buffer park established in 2001 and subsequent ones established mostly in the 2010s (Otterman 2015). This history of disturbance, coupled with the recency of establishment for these buffer parks, thus suggest that buffer streams may have experienced high levels of historical disturbance.

In the current context, buffer parks are slated for forest restoration efforts (Suppl. material 1: fig. S7) under the Forest Restoration Action Plan (NParks 2019). Depending on the nature park, interventions adopted may include tree and shrub planting, removal of weeds and fast-growing plants, and stream restoration which involves planting of riverine vegetation along stream banks (Tan et al. 2023). Open all-year round, buffer parks also receive constant public visitation during daylight hours (NParks 2024), with some of these streams situated near hiking trails, unlike in nature reserves where streams tend to be situated further from hiking trails, or even in areas closed to public access (Chan, Y.Q., pers. observ.). Heavy usage of hiking trails results in increased soil erosion and, over time, soil compaction, which causes greater runoff into streams (Salesa and Cerdà 2020). The resulting physico-chemical changes that likely occur include increased turbidity, TDS, and nutrients, which affect Trichoptera larval assemblages (Kalaninová et al. 2014). In the present study, higher TDS, conductivity, and salinity were observed in buffer streams when compared against forest streams, along with differences in observed Trichoptera diversity between stream types.

Continuous disturbance from these various sources likely prevents the establishment of climax communities, causing stream communities to alternate between establishment and recovery (Death 1996). Human disturbance may also allow for greater co-existence of species between rapid coloniser species and highly competitive species, preventing the dominance of either group (Wilson 1994), which following the Intermediate Disturbance Hypothesis (Mayor et al. 2012), may account for the highest diversity found in sampled buffer streams.

### ﻿Absence of Trichoptera in urban streams

In this study, no Trichoptera were found in the two urban streams surveyed. Urban streams are often associated with poor water quality, and thus lower abundance of intolerant taxa (Hughes 2006; Urban et al. 2006; Violin et al. 2011). The PCA in the present study also revealed that urban sites were associated with higher TDS, which is often a result of anthropogenic input from runoff (Köse et al. 2014), suggesting that the sampled urban streams experience a greater extent of pollution and anthropogenic disturbance (da Silva et al. 2020; Correa-Araneda et al. 2022) than buffer and forest streams. Using SingScore, a macroinvertebrate biotic index developed for Singapore streams, the assigned scores for the six families collected in the present study range from 6–10, suggesting medium to low pollution tolerance (cf., the full range of SingScore-Tolerance scores from 1 (most pollution-tolerant) to 10 (least pollution-tolerant)) (Blakely et al. 2014). Therefore, the absence of Trichoptera from urban streams, coupled with higher TDS, may indicate poorer water quality relative to buffer and forest streams and aligns with the findings by Wiederkehr et al. (2020).

### ﻿No significant difference between buffer and forest streams

Similarities in environmental conditions and Trichoptera assemblages were observed between buffer and forest streams. Taxonomic and functional structure of Trichoptera assemblages are strongly associated with stream order and other environmental conditions such as temperature, flow, and substrate type (Huryn and Wallace 1988; Lamouroux et al. 2004; Hughes 2006; Kalaninová et al. 2014). Hence, given the similarity of environmental conditions between buffer and forest streams, their trichopteran assemblages could be expected to be similar as well.

The similarities in Trichoptera assemblages could also be due to Singapore’s geographical features, with a relatively small area, a generally flat topography, and a large proportion of land area being urbanised (Chia et al. 1991). This results in a compression of stream orders (Wiederkehr et al. 2020) such that most of Singapore’s naturally occurring streams are lower order and therefore ecologically similar in that characteristic. Furthermore, buffer parks in Singapore are close to nature reserves, being situated along their peripheries (NParks 2024). This proximity increases the likelihood of movement between buffer parks and nature reserves, aiding in adult Trichoptera dispersal across stream types (Curry and Baird 2015) and widening the range of the organisms (NParks 2015). Additionally, buffer and forest streams are often situated within the same drainage basins, potentially supporting a metacommunity of Trichoptera families in a single basin across buffer parks and nature reserves (Brown and Swan 2010), further explaining why similar Trichoptera assemblages are observed across these two stream types.

### ﻿Comparison with past studies in Singapore

The six Trichoptera families collected in the present study are a subset of the 18 families found in Peninsular Malaysia and Singapore (Morse 2004). Within Singapore, only two recent studies have documented Trichoptera larvae (Ho et al. 2018; Wiederkehr et al. 2020).

A study on aquatic macroinvertebrate diversity in Nee Soon Swamp Forest (NSSF), Singapore’s only remaining freshwater swamp forest, recorded 11 families, with Ecnomidae as the most abundant family (Ho et al. 2018). Trichoptera were collected during 2013–2015 using methods similar to that of the present study, except for an overall higher sampling effort owing to many more sampling sites within NSSF, covering more diverse microhabitats (*N* = 40). Additionally, Ho et al. (2018) discussed the characteristic waterlogged, ‘freshwater swamp forest’ stream environmental conditions within NSSF – lower flow, higher tannins, and higher acidity – as opposed to faster-flowing forest streams with visibly less tannins, which were sampled in the present study, and excluded NSSF streams. These environmental conditions may potentially account for the high diversity of Trichoptera (Lamouroux et al. 2004; Hughes 2006; Kalaninová et al. 2014) in that study; however, this can only be confirmed by further analysis of the environmental conditions of sampling sites and their corresponding trichopteran assemblages within NSSF. There also exists high heterogeneity within NSSF, comprising both lentic and lotic freshwater habitats, in addition to periodic reservoir freshwater input in the northeast edge (Ho et al. 2018)—in contrast to the present study, which focused only on lotic environments without reservoir input. Given the specificity of niches filled by various Trichoptera families (Morse 2004; Holzenthal et al. 2015), the wider range of freshwater habitats covered as well as the greater sampling effort by Ho et al. (2018) within NSSF, might thus account for the higher Trichoptera diversity reported in comparison to the present study.

Another study investigating macroinvertebrates across 11 urban, buffer, and forest streams in Singapore (Wiederkehr et al. 2020) recorded Trichoptera in all three stream types; however, diversity was limited to only two families, Ecnomidae and Polycentropodidae. Polycentropodidae were recorded in relatively low abundances per site (0–3.8; Wiederkehr et al. 2020), and this is comparable to the present study (0–1 Polycentropodidae per site). Ecnomidae was the more abundant family among the two recorded by Wiederkehr et al. (2020; similar to NSSF by Ho et al. 2018), contrasting with the present study, which found no Trichoptera in sampled urban streams and Ecnomidae to be the least abundant family. This could be attributed to the difference in collection method used by Wiederkehr et al. (2020), which made use of leaf-litter bags that selectively attract leaf-litter dwelling macroinvertebrates. Given the passive and targeted nature of that collection method, the two Trichoptera families collected in the study by Wiederkehr et al. (2020) are likely not as representative of the entire stream Trichoptera assemblage. In the present study, the six families collected comprise a variety of functional feeding groups, including filtering-collectors and facultative predators (Ecnomidae, Hydropsychidae, Polycentropodidae), obligate predators (some Leptoceridae), shredding detritivores (most Calamoceratidae, most Leptoceridae, Odontoceridae), and scrapers (some Calamoceratidae). Furthermore, collected families in the present study differed between case-making larvae that build portable cases of plant matter (Calamoceratidae, some Leptoceridae) or minerals (some Leptoceridae, Odontoceridae) and fixed-retreat-making larvae (Ecnomidae, Hydropsychidae, Polycentropodidae), the fixed retreats suggesting a preference for harder sediment types (Morse 2004). These two reasons could further explain why a greater diversity of Trichoptera was found in the present study than in the study by Wiederkehr et al. (2020).

## ﻿Conclusion

Overall, the present study has confirmed the continued presence of Trichoptera in Singapore, providing a snapshot of Singapore’s Trichoptera diversity and distribution within the Northeast Monsoon months of October 2023 to January 2024. Differences in Trichoptera assemblages and environmental conditions were observed across an urbanisation gradient, comprising forest, buffer, and urban streams, in which Trichoptera were recorded as being absent from urban streams. This thus affirms the effectiveness of current freshwater conservation efforts by NParks, while also emphasising the need to continue managing urbanisation impacts to allow such pollution-sensitive and diverse taxa to continue existing. Future work could thus be done to investigate temporal trends in Trichoptera diversity, which would shed light on Trichoptera dispersal patterns that will be important to inform conservation efforts of this taxa. Additionally, taxonomic work identifying Trichoptera specimens to genus or species would also be useful to deepen current knowledge of Trichoptera diversity in Singapore.

## ﻿Additional information

### Use of AI

No use of AI was reported.

### Author ORCIDs

Yee Qi Chan https://orcid.org/0009-0005-9683-3596

Yuchen Ang https://orcid.org/0000-0001-5889-018X

John C. Morse https://orcid.org/0000-0003-3187-4045

Yixiong Cai https://orcid.org/0009-0008-6499-5795

Darren C. J. Yeo https://orcid.org/0000-0003-2669-4349

### Data availability

All of the data that support the findings of this study are available in the main text or Supplementary Information.

## Supplementary materials

Supplementary material 1 Supplementary tables and figures Data type odt https://binary.pensoft.net/file/1488023This dataset is made available under the Open Database License (http://opendatacommons.org/licenses/odbl/1.0/). The Open Database License (ODbL) is a license agreement intended to allow users to freely share, modify, and use this Dataset while maintaining this same freedom for others, provided that the original source and author(s) are credited.Yee Qi Chan, Bryna Jia Ying Liang, Yuchen Ang, John C. Morse, Yixiong Cai, Darren C. J. Yeo
