# Supplementary material for: ﻿Diversity, abundance and distribution of caddisfly (Insecta, Trichoptera) families in relation to environmental parameters across freshwater streams in Singapore
Source: Zookeys. 2025 Dec 10;1263:21–36. doi: 10.3897/zookeys.1263.147968 (PMC12712629; doi:10.3897/zookeys.1263.147968)
Supplement: Supplementary file 1 [file zookeys-1263-021_article-147968__-s001.odt]

# Supplementary Material

# Table S1. Sampling sites across stream types (forest, buffer & urban streams).

| # Forest stream | # Taban StreamA muddy path in the middle of a forest  Description automatically generated | # Butterfly TrailA dirt path with trees and plants  Description automatically generated | # Upper PeirceA stream in the woods  Description automatically generated | # Petaling StreamA muddy path in the middle of a forest  Description automatically generated | # CCNR U-streamA dirt path in the middle of a forest  Description automatically generated |
| --- | --- | --- | --- | --- | --- |
| # Buffer stream | # Wallace StreamA stream running through a forest  Description automatically generated | # Thomson Nature ParkA dirt path with trees and plants  Description automatically generated | # Windsor Nature ParkA stream in the middle of a forest  Description automatically generated | # Asas StreamA stream of water with a stream running through it  Description automatically generated |  |
| # Urban stream | # Lorong ChencharuA river flowing through a forest  Description automatically generated | # Clementi ForestA stream running through a forest  Description automatically generated |  |  |  |

| Table S2. Table of environmental parameters across forest and buffer streams. The table shows mean ± SE and p-value indicating significance of difference between buffer and forest streams, if any. |
| --- |
| # | # Buffer (mean ± SE) | # Forest (mean ± SE) | # p-value |
| # Surface flow rate (km/h) | # 1.43 ± 0.73 | # 1.19 ± 0.36 | # 0.730 |
| # Hardness | # 1.75 ± 0.49 | # 1.20 ± 0.39 | # 0.157 |
| # Temperature (°C) | # 26.00 ± 0.24 | # 26.04 ± 0.20 | # 0.802 |
| # Wetted depth (cm) | # 6.66 ± 2.39 | # 6.88 ± 3.90 | # 1 |
| # Wetted width (cm) | # 222.8 ± 91.4 | # 305.6 ± 167.1 | # 0.623 |
| # Dissolved oxygen, DO (mg/L) | # 6.71 ± 0.55 | # 6.08 ± 0.81 | # 0.286 |
| # Conductivity (μS/cm) | # 62.73  ± 36.7 | # 32.84  ± 12.4 | # 0.286 |
| # Total dissolved solids (mg/L) | # 39.98 ± 23.05 | # 9.05 ± 7.93 | # 0.219 |
| # Salinity (ppm) | # 0.028 ± 0.016 | # 0.012 ± 0.003 | # 0.106 |
| # pH | # 6.05 ± 0.78 | # 5.32 ± 0.49 | # 0.191 |
| # Canopy cover (%) | # 88.9 ± 10.2 | # 91.7 ± 5.21 | # 0.905 |
| # Count | # 17.3 ± 13.4 | # 7.2 ± 4.4 | # 0.387 |
| # Population density (m-2) | # 0.68 ± 0.29 | # 0.32 ± 0.19 | # 0.178 |

A shelf with containers of food

Description automatically generated

Figure S1. Collected and sieved sediment stored in labelled, plastic jars filled with 96% ethanol. 

A close up of a white square with brown specks

Description automatically generated

Figure S2. Visual sorting of macroinvertebrates (left) from sediment in trays, (right) with a close-up of an identified Trichoptera larva in its case.

A close-up of a shrimp

Description automatically generated

Figure S3. Leptoceridae larva removed from its case. 

A close-up of a bug

Description automatically generated

Figure S4. Larval characteristics used to identify Trichoptera larvae to family level.

A diagram of different types of bacteria

Description automatically generated

Figure S5. A non-metric dimensional scaling (NMDS) plot. Trichoptera assemblages are compared across forest (in green) and buffer (in yellow) streams, based on the Bray-Curtis distance (stress = 0.055). The 95% confidence ellipse for both stream types are plotted, and individual sites are represented by dots.

A group of boxes with different colored squares

Description automatically generated

Figure S6. Boxplots of Shannon diversity, richness and abundance across buffer (in yellow) and forest (in green) streams. Hinges show the mean ± SE, and the upper and lower whiskers show the maximum and minimum value, respectively. The corresponding p-values from Mann-Whitney U-tests are also shown at the top of each plot.

Forest Restoration sites

Figure S7. Forest restoration sites (light green). These are located within buffer parks around Bukit Timah Nature Reserve and Central Catchment Nature Reserve under the Forest Restoration Action Plan (NParks 2019).
